# Comparative Analysis of Carbon Monoxide Tolerance among *Thermoanaerobacter* Species

**DOI:** 10.3389/fmicb.2016.01330

**Published:** 2016-08-29

**Authors:** Joana I. Alves, M. Madalena Alves, Caroline M. Plugge, Alfons J. M. Stams, Diana Z. Sousa

**Affiliations:** ^1^Centre of Biological Engineering, University of MinhoBraga, Portugal; ^2^Laboratory of Microbiology, Wageningen UniversityWageningen, Netherlands

**Keywords:** syngas, carbon monoxide, *Thermoanaerobacter*, sugar fermentation, ethanol, hydrogen

## Abstract

An anaerobic thermophilic strain (strain PCO) was isolated from a syngas-converting enrichment culture. Syngas components cannot be used by strain PCO, but the new strain is very tolerant to carbon monoxide (pCO = 1.7 × 10^5^ Pa, 100% CO). 16S rRNA gene analysis and DNA-DNA hybridization revealed that strain PCO is a strain of *Thermoanaerobacter thermohydrosulfuricus*. The physiology of strain PCO and other *Thermoanaerobacter* species was compared, focusing on their tolerance to carbon monoxide. *T. thermohydrosulfuricus, T. brockii* subsp. *finnii, T. pseudethanolicus*, and *T. wiegelii* were exposed to increased CO concentrations in the headspace, while growth, glucose consumption and product formation were monitored. Remarkably, glucose conversion rates by *Thermoanaerobacter* species were not affected by CO. All the tested strains fermented glucose to mainly lactate, ethanol, acetate, and hydrogen, but final product concentrations differed. In the presence of CO, ethanol production was generally less affected, but H_2_ production decreased with increasing CO partial pressure. This study highlights the CO resistance of *Thermoanaerobacter* species.

## Introduction

Thermophiles thrive at austere and unusual conditions and their evolutionary significance and biotechnological potential have triggered microbiological research over the last decades (Turner et al., 2007; Wagner and Wiegel, 2008; Yoneda et al., 2015). There is a continuous biotechnological interest in highly thermostable enzymes, which make this kind of organisms very attractive. Thermophilic bacteria of the class Clostridia, such as members of the genera *Clostridium, Thermoanaerobacter, Thermoanaerobacterium*, and *Caldicellulosiruptor*, are currently used as biocatalysts for the production of biofuels or other chemicals of interest (Hemme et al., 2010; Carere et al., 2012). Specifically members of the *Thermoanaerobacter* genus are utilized to produce ethanol and hydrogen (H_2_) from a variety of saccharides (Jessen and Orlygsson, 2012). Within thermophiles, an organism from *Thermoanaerobacter* genus—*T. ethanolicus*—is one of the most well-studied ethanol-producing bacteria (Wiegel and Ljungdahl, 1981; Lacis and Lawford, 1991). A less common substrate, carbon monoxide (CO), is used by *T. thermohydrosulfuricus* subsp. *carboxydovorans* and *T. kivui*. *T. thermohydrosulfuricus* subsp. *carboxydovorans* can grow with CO as sole electron donor (25% in the headspace), producing H_2_ and CO_2_ (Balk et al., 2009). *T. thermohydrosulfuricus* shares 99% similarity of the 16S rRNA gene sequence and over 70% DNA-DNA hybridization with *T. thermohydrosulfuricus* subsp. *carboxydovorans*, but only the latter one can use CO. Growth of the homoacetogenic *T. kivui* with CO diluted with CO_2_/N_2_ or CO_2_/H_2_ was described by Kevbrina et al. (1996). Recently, Weghoff and Müller (2016) reported the ability of *T. kivui* to grow on only CO (100% in the headspace), producing acetate and hydrogen. Carboxydotrophic metabolism in *Thermoanaerobacter* species is normally not assessed, and it is not known if they can endure CO or even adapt to grow on CO, as recently reported for *T. kivui* (Weghoff and Müller, 2016). In this work we isolated *Thermoanaerobacter thermohydrosulfuricus* strain PCO from a thermophilic syngas-converting enrichment, but this strain appears unable to oxidize CO. The main objectives of this work were (1) to characterize and determine the CO tolerance of *Thermoanaerobacter thermohydrosulfuricus* strain PCO, and (2) to compare the effect of CO on growth, glucose consumption and product formation of strain PCO and of four close relative species from the *Thermoanaerobacter* genus.

## Materials and methods

### Enrichments and isolation

Suspended sludge from a thermophilic anaerobic municipal solid waste digester (Barcelona, Spain) was used as inoculum for starting up syngas-converting enrichments. Microbial cultures were enriched with synthetic syngas (mixture of 60% CO, 10% CO_2_, and 30% H_2_, total pressure 1.7 × 10^5^ Pa) as sole carbon and energy source (Alves et al., 2013). Isolation of strain PCO was done using soft agar (1.5%, w/v) incubations and liquid medium serial dilutions, with 20 mM pyruvate as sole substrate. Sodium pyruvate was added to the medium from a 1M filter-sterilized stock solution. A phosphate-buffered mineral medium was used, containing (per liter): Na_2_HPO_4_, 1.63 g; NaH_2_PO_4_, 1.02 g; resazurin, 0.5 g; NH_4_Cl, 0.3 g; CaCl_2_·2H_2_O, 0.11 g; MgCl_2_·6H_2_O, 0.10 g; NaCl, 0.3 g; 1 mL of acid and alkaline trace element stock each, and 0.2 ml of vitamin stock. Trace elements and vitamins were prepared as described previously (Stams et al., 1993). Before inoculation, medium was reduced with sodium sulfide (0.8 mM final concentration). Bottles were incubated in the dark at 55°C while shaken at 100 rpm (liquid cultures) or standing (soft-agar cultures). Colonies were picked from soft-agar incubations, inoculated in fresh liquid medium containing pyruvate (20 mM). Cultures were further purified by subsequent serial dilutions alternating with soft-agar colony picking. Purity of the culture was checked by microscopic examination after growth with different substrates (Olympus CX41, Tokyo, Japan). Direct sequencing of the 16S rRNA gene and denaturing gradient gel electrophoresis (DGGE) were also applied to check the genetic purity of the culture.

### DNA isolation, PCR and DGGE

Genomic DNA from strain PCO was extracted using the FastDNA SPIN kit for soil (MP Biomedicals, Solon, OH), according to the manufacturer's instructions. The 16S rRNA gene was directly amplified from genomic DNA by PCR, using the primer set 027F/1492R (Nübel et al., 1996) and the following PCR program: pre-denaturation, 2 min at 95°C; 30 cycles of denaturation, 30 s at 95°C, annealing, 40 s at 52°C, and elongation, 90 s at 72°C; and post-elongation, 5 min, at 72°C. For DGGE analysis, the 16S rRNA gene was partially amplified from genomic DNA with primer set U968GC-f/L1401-r (Lane, 1991; Muyzer et al., 1993). The thermocycling program used for PCR-DGGE amplification was: pre-denaturation, 5 min at 95°C; 35 cycles of denaturation, 30 s at 95°C, annealing, 40 s at 56°C, and elongation, 90 s at 72°C; and post-elongation, 5 min at 72°C. DGGE was performed using a DCode system (Bio-Rad, Hercules, CA). Gels contained 8% (wt/vol) polyacrylamide (37.5:1 acrylamide/bis-acrylamide) and a linear denaturing gradient of 30–60%, with 100% of denaturant corresponding to 7 M urea and 40% (vol/vol) formamide. Electrophoresis was performed for 16 h at 85 V and 60°C in a 0.5x Tris-Acetate–EDTA buffer. DGGE gels were stained with silver nitrate (Sanguinetti et al., 1994).

### Sequencing and phylogenetic analysis

PCR products obtained from 16S rRNA gene amplification were purified using the PCR Clean Up kit NucleoSpin Extract II (Macherey-Nagel, Düren, Germany) and sequenced directly at Eurofins MWG Operon (Ebersberg, Germany). Partial sequences were assembled using the alignment editor BioEdit v7.0.9 software package (Hall, 1999). Similarity searches for the 16S rRNA gene sequence derived from strain PCO were performed using the NCBI BLAST search program within the GenBank database (Altschul et al., 1990). The 16S rRNA gene sequence of *Thermoanaerobacter* strain PCO is available in the DDBL/EMBL/GenBank databases under the accession number HF586422.

### Characterization of strain PCO and cultivation of *Thermoanaerobacter* strains

Unless otherwise stated, all the physiological tests of strain PCO and its close relatives (*T. thermohydrosulfuricus, T. brockii* subsp. *finnii, T. pseudethanolicus*, and *T. wiegelii*) were performed using a bicarbonate-buffered mineral salt medium (Stams et al., 1993). Type strains of *Thermoanaerobacter thermohydrosulfuricus* (DSM 527^T^), *T. brockii* subsp. *finnii* (DSM 3389^T^), *T. pseudethanolicus* (DSM 2355^T^), and *T. wiegelii* (DSM 10319^T^) were obtained from the Deutsche Sammlung von Mikroorganismen und Zellkulturen (DSMZ; German Collection of Microorganisms and Cell Cultures, Braunschweig, Germany). Growth of strain PCO was tested with the following substrates (at a concentration of 20 mM unless indicated otherwise): acetate, arabinose, cellobiose, cellulose (5 g L^−1^), ethanol, formate, fructose, galactose, glucose, glycerol, glycine, lactate, lactose, maltose, mannitol, mannose, methanol, pectin (5 g L^−1^), propionate, pyruvate, raffinose, ribose, sorbitol, starch (5 g L^−1^), sucrose, trehalose, xylan (5 g L^−1^), xylose, yeast extract (5 g L^−1^), CO (from 20 to 100% CO, 1.7 × 10^5^ Pa), and H_2_/CO_2_ (80/20%, 1.7 × 10^5^ Pa). Utilization of different electron acceptors (elemental sulfur, AQDS, sulfate, sulfite, thiosulfate, nitrate, and nitrite) by strain PCO was done using pyruvate (20 mM) as electron donor, while glucose (20 mM) was for tests with *T. thermohydrosulfuricus, T. brockii* subsp. *finnii, T. pseudethanolicus*, and *T. wiegelii*. Pyruvate (20 mM) was used to test the optimum growth temperature (range 20–85°C) and pH (range 5.7–8.0) of strain PCO. All the assays were done in duplicate. Cell growth was determined by measuring optical density at 600 nm with a spectrophotometer (U-1500 Hitachi, Tokyo, Japan). Cell morphology of strain PCO was examined by phase contrast microscopy (Leica DM 2000, Wetzlar, Germany). Cells from active cultures of strain PCO were stained using standard Gram staining techniques. The DNA–DNA hybridization analysis and the G+C content of the DNA were determined by the identification service of the Deutsche Sammlung von Mikroorganismen und Zellkulturen (DSMZ; German Collection of Microorganisms and Cell Cultures, Braunschweig, Germany).

### Carbon monoxide tolerance tests

Strain PCO, *Thermoanaerobacter thermohydrosulfuricus, T. brockii* subsp. *finnii, T. pseudethanolicus*, and *T. wiegelii* were tested for CO tolerance. All cultures were incubated with 0, 50, and 100% CO (pCO/P, where “pCO” is the CO partial pressure and “P” the total gas pressure). Additionally, strain PCO was incubated with 25 and 75% CO. Initial total pressure was 1.7 × 10^5^ Pa in all the assays; N_2_ was used to pressurize the headspace for CO percentages lower than 100%. The tests were performed using an anaerobic phosphate-buffered mineral salt medium and glucose (20 mM) was used as carbon and energy source. Bottles were incubated in the dark, at 55°C and shaken at 100 rpm. All the assays were done in duplicate. Growth of the strains was determined by measuring optical density increase at 600 nm with a spectrophotometer (U-1500 Hitachi, Tokyo, Japan). The statistical significance of the differences detected in glucose conversion rates and end products production was evaluated using single factor analysis of variances (ANOVA).

### Analytical methods

Soluble substrates and intermediates (sugars, organic acids, and alcohols) were measured using a HPLC Thermo Electron equipment with a Shodex SH1821 column and equipped with a RI detector. The mobile phase used was sulfuric acid (0.01 N) at a flow rate of 0.6 mL min^−1^. Column temperature was set at 60°C. Inorganic anions were analyzed by chromatography using a HPLC Dionex system, equipped with an Ionpac AS22 column, and ED40 electrochemical detector. Column temperature and pressure varied between 35–40°C and 130 × 10^5^–160 × 10^5^ Pa. Gaseous compounds (CO, CO_2_, H_2_) were analyzed by gas chromatography on a GC-2014 Shimadzu with a thermal conductivity detector. CO_2_ was analyzed with a CP Poraplot Q column (25 m length, 0.53 mm internal diameter; film thickness, 20 μm). Helium was used as carrier gas at a flow rate of 15 mL min^−1^, and the temperatures in the injector, column, and detector were 60, 33, and 130°C. CO and H_2_ were analyzed with a Molsieve 13X column (2 m length, 3 mm internal diameter). Argon was used as carrier gas at a flow rate of 50 mL min^−1^, and temperatures in the injector, column, and detector were 80, 100, and 130°C.

## Results and discussion

### Physiological characterization of strain PCO and comparison with closely related *Thermoanaerobacter* species

**Figure 1:**
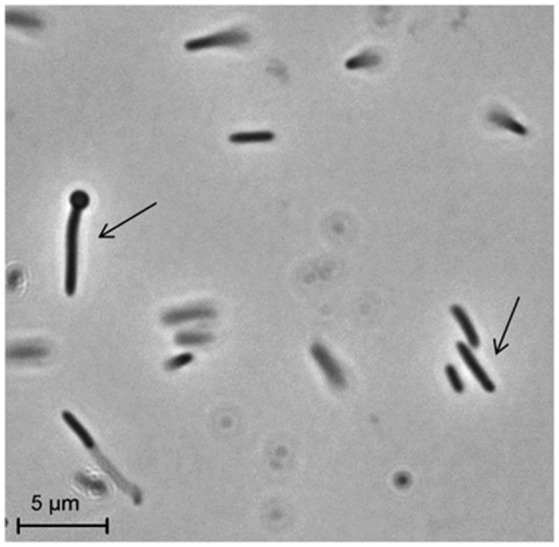
**Phase-contrast micrographs showing morphology of cells of strain PCO**. The arrows indicate vegetative and sporulating cells. Bar, 5 μm.

**Figure 2:**
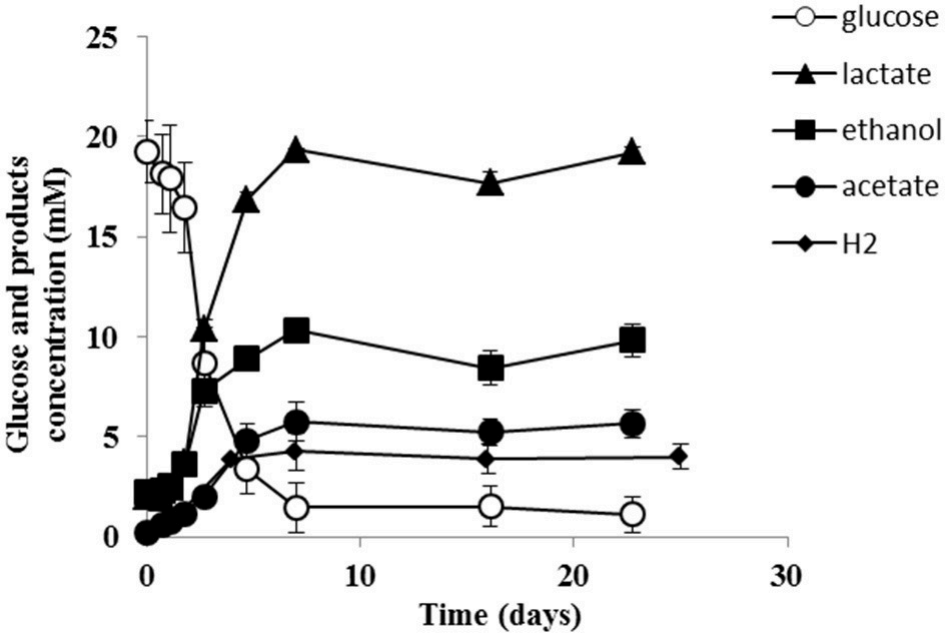
**Glucose conversion and products formation by strain PCO over time**. The results represent the average of duplicate experiments.

Strain PCO was isolated from the thermophilic syngas-converting enrichment described by Alves et al. (2013). Isolation was performed using pyruvate as sole carbon and energy source. Strain PCO has a G+C content of the DNA of 34.5 mol % and shares 98% identity with the 16S rRNA gene of *Thermoanaerobacter thermohydrosulfuricus* (the 16S rRNA gene sequence of strain PCO is available in the DDBL/EMBL/GenBank databases under the accession number HF586422). DNA-DNA hybridization between the two strains was 100%, confirming that strain PCO is a strain of *Thermoanaerobacter thermohydrosulfuricus*. Strain PCO was not a predominant microorganism in the syngas enriched culture, as shown by the molecular characterization (Alves et al., 2013). However, its presence indicates that it can grow on metabolic byproducts and/or dead cells at high CO concentrations. Strain PCO formed terminal round endospores, which is a characteristic of *Thermoanaerobacter* species (Wiegel and Ljungdahl, 1981; Lee et al., 1993, 2007; Kim et al., 2001; Balk et al., 2009; Shaw et al., 2010). Cells of strain PCO are straight rods and normally occur singly (Figure 1). Strain PCO had an optimum growth temperature of 70°C; no growth was detected below 37°C or above 75°C. The optimum pH for growth was between 6.5 and 7.5. Strain PCO is a very versatile organism that can utilize a range of different substrates, such as: arabinose, cellobiose, cellulose, fructose, galactose, glucose, lactose, maltose, mannitol, mannose, pectin, pyruvate, raffinose, ribose, sorbitol, starch, sucrose, trehalose, xylan, xylose, and yeast extract. No growth occurred with acetate, ethanol, formate, glycerol, glycine, lactate, methanol, propionate, CO (from 20 to 100% CO, total pressure 1.7 × 10^5^ Pa), and H_2_/CO_2_ (80/20%, total pressure 1.7 × 10^5^ Pa). The main products detected and quantified from glucose fermentation by strain PCO were lactate, ethanol, acetate, and H_2_ (Figure 2) which are typically formed from glucose by most of the *Thermoanaerobacter* species (Wiegel and Ljungdahl, 1981; Lee et al., 1993; Kim et al., 2001; Lee et al., 2007; Balk et al., 2009; Shaw et al., 2010). Strain PCO is able to reduce elemental sulfur and AQDS, but sulfate, sulfite, thiosulfate, nitrate, and nitrite could not serve as electron acceptors. The comparison between the morphological, biochemical and physiological characteristics of strain PCO and its close relatives is presented in Table 1. All of them can use thiosulfate as electron acceptor, but strain PCO cannot. Even though strain PCO and *T. thermohydrosulfuricus* are the same species, strain PCO can be differentiated because of its ability to grow and ferment cellulose and reduce AQDS.

**Table 1:** **Physiological and biochemical characteristics of (1) strain PCO and its phylogenetic related species: (2) ***Thermoanaerobacter thermohydrosulfuricus*** (DSM 527^**T**^), (3) ***T. brockii*** subsp. ***finnii*** (DSM 3389^**T**^), (4) ***T. pseudethanolicus*** (DSM 2355^**T**^), and (5) ***T. wiegelii*** (DSM 10319^**T**^)**.

**Characteristics**	1^*^	**2**	**3**	**4**	**5**
Growth pH (optimum)	6.5–7.5	6.9–7.5	6.5–6.8	nr	6.8
Growth temperature (optimum) (°C)	70	67–69	65	65	65–68
Spore formation	+	+	+	+	+
Gram reaction	Negative	Variable	Variable	Variable	Negative
DNA G+C content (mol %)	34.5	37.6	32	34.4	35.6
**SUBSTRATE UTILIZATION**					
Arabinose	+	±	−^*^	−	−
Carboxymethylcellulose	±	+	−^*^	−^*^	+^^*^^
Cellobiose	+	+	+	+	+
Cellulose	+	−	±^*^	−^*^	+^*^
Acetate	−	−	−^*^	−^*^	−^*^
Fructose	+	+	+	−^*^	+
Galactose	+	+	+	+^*^	+
Glucose	+	+	+	+	+
Lactose	+	+^*^	+	−^*^	+
Maltose	+	+	+	+	+
Mannose	+	+	+	±^*^	+
Raffinose	+	+^*^	+^*^	+^*^	+
Ribose	+	+	+	+	−
Sucrose	+	+	+	+	+
Trehalose	+	+	+^*^	+^*^	+^*^
Xylose	+	+	+	+	+
Starch	+	+	+^*^	+	+
Pectin	+	+	±^*^	−^*^	+
Peptone	±	+	−^*^	±^*^	+^*^
Xylan	+	+	+^*^	+^*^	+^*^
Yeast extract	+	+	−^*^	−^*^	+^*^
Pyruvate	+	+	+	+	−
Ethanol	−	−^*^	−^*^	−^*^	−
Glycerol	−	−	±^*^	−	+
Mannitol	+	±	+	−	+
Methanol	−	−^*^	−^*^	−^*^	−^*^
Sorbitol	+	+^*^	±^*^	±^*^	+^*^
CO	−	−	−^*^	−^*^	−^*^
H_2_/CO_2_	−	−^*^	−^*^	−	−^*^
Formate	−	−^*^	−^*^	−^*^	−^*^
Glycine	−	±^*^	−^*^	−^*^	±^*^
Lactate	−	−	−^*^	−^*^	−
Propionate	−	−^*^	−^*^	−^*^	−^*^
Succinate	−	−^*^	−^*^	−^*^	−
**ELECTRON ACCEPTORS**					
AQDS	+	−^*^	+^*^	+^*^	+^*^
Elemental sulfur	+	+^*^	+^*^	+^*^	+^*^
Nitrate	−	+	−^*^	−^*^	−^*^
Nitrite	−	−	−^*^	−^*^	−^*^
Sulfate	−	−	−^*^	−^*^	−^*^
Sulfite	−	+	−^*^	−^*^	+^*^
Thiosulfate	−	+	+	+	+^*^

Data are from Cayol et al. (1995), Lee et al. (1993), Onyenwoke et al. (2007), Cook et al. (1996) and this study. nr, not reported. Symbols: +, utilized; ±, poorly utilized; −, not utilized;

* *, data from this study*.

### Carbon monoxide tolerance of strain PCO and related *Thermoanaerobacter* sp.

**Figure 3:**
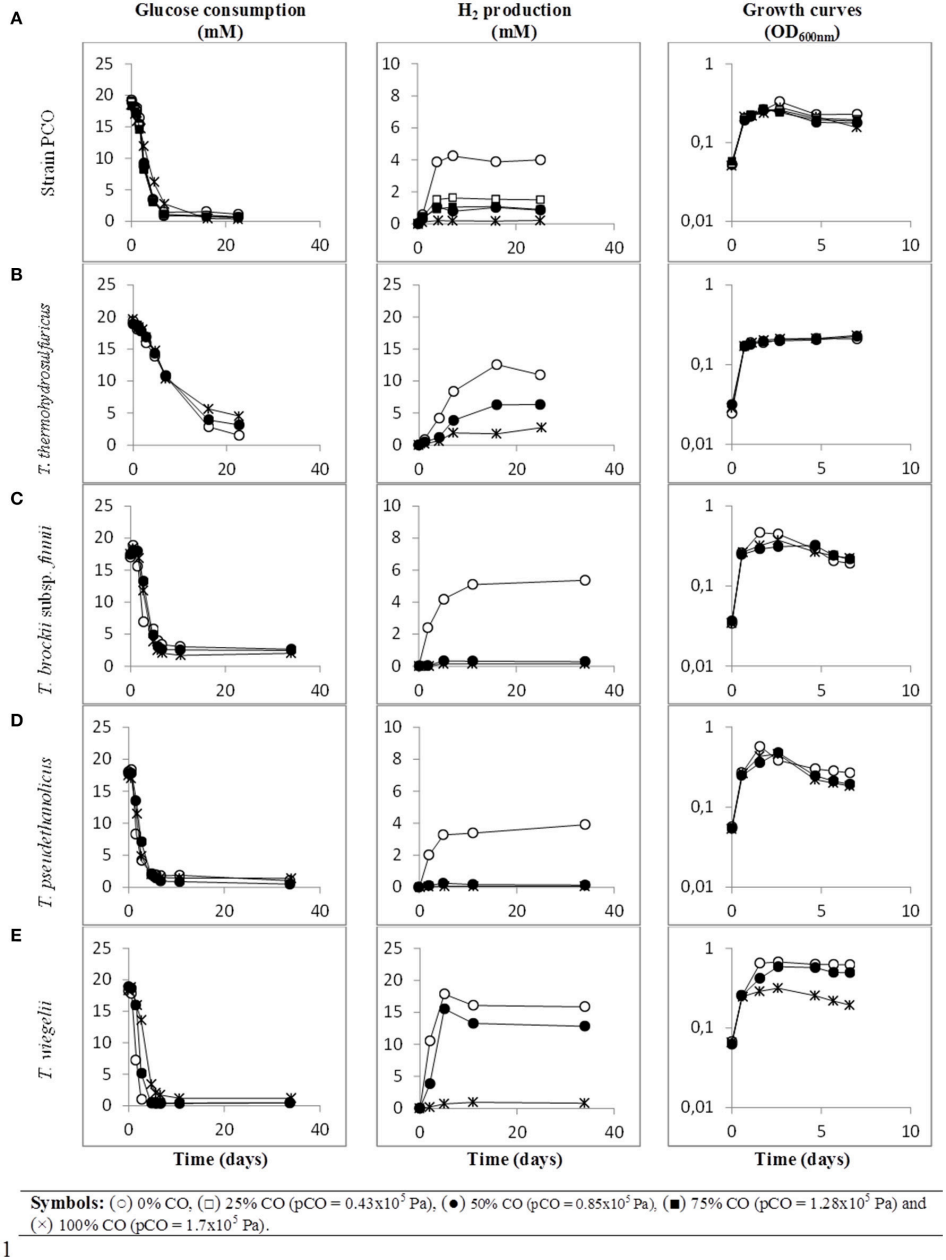
**Glucose conversion, production of H_**2**_ from glucose and growth curves over time by (A) strain PCO, (B) ***Thermoanaerobacter thermohydrosulfuricus*** (DSM 527^**T**^), (C) ***T. brockii*** subsp. ***finnii*** (DSM 3389^**T**^), (D) ***T***. ***pseudethanolicus*** (DSM 2355^**T**^), and (E) ***T. wiegelii*** (DSM 10319^**T**^), in incubations with different CO concentration in the gas phase (0, 25, 50, 75, or 100% CO)**. Plotted are the average data of duplicate experiments. The values of optical density were plotted vs. time on a logarithmic scale.

**Figure 4:**
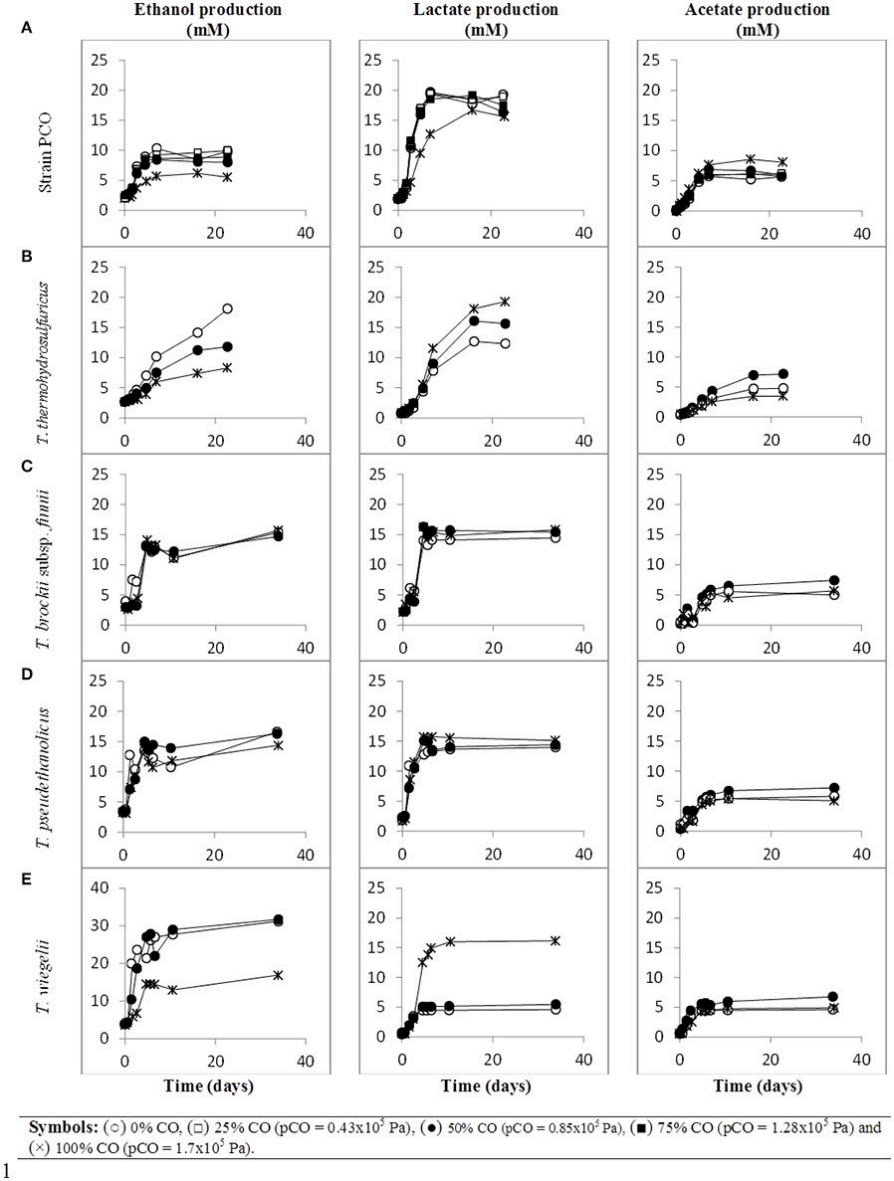
**Production of ethanol and organic acids (lactate and acetate) from glucose, over time, by (A) strain PCO, (B) ***Thermoanaerobacter thermohydrosulfuricus*** (DSM 527^**T**^), (C) ***T. brockii*** subsp. ***finnii*** (DSM 3389^**T**^), (D) ***T***. ***pseudethanolicus*** (DSM 2355^**T**^), and (E) ***T. wiegelii*** (DSM 10319^**T**^), in incubations with different CO concentration in the gas phase (0, 25, 50, 75, or 100% CO)**. Plotted are the average data of duplicate experiments.

When strain PCO was cultured with 20 mM of glucose and subjected to different CO concentrations in the headspace (0, 25, 50, 75, and 100%, total pressure 1.7 × 10^5^ Pa) no significant differences were found in glucose consumption rates (Table 2). Carbon monoxide concentrations of 75% or lower did not affect substantially product formation from glucose (Figures 3A, 4A), except for H_2_. Hydrogen production by strain PCO decreased significantly (*P* = 0.0001) from 3.94 ± 0.55 to 1.52 ± 0.13 mM when cultures were incubated with 0 and 25% CO in the headspace, respectively (Figure 3A). When testing different CO partial pressures, from 25 to 100%, a decrease in H_2_ production is observed, with H_2_ production of < 0.02 mmol L^−1^ at 100% CO. The other end products from glucose conversion were only affected when strain PCO was exposed to 100% CO. There was a significant decrease on the final production of lactate (*P* = 0.01) and ethanol (*P* = 0.004) only when comparing cultures grown with 75 and 100% CO (Figure 4A). These results could be explained due to the fact that this strain had been isolated from a syngas-converting culture, that was in contact with high CO concentrations for over 1 year (Alves et al., 2013), which might have increased the tolerance of strain PCO to CO. There are only two *Thermoanaerobacter* species able to use CO: *T. thermohydrosulfuricus* subsp. *carboxydovorans* and *T. kivui* (Balk et al., 2009; Weghoff and Müller, 2016); for other *Thermoanaerobacter* species the ability to convert CO was never reported. The ability to utilize CO as carbon and energy source and/or the ability to tolerate CO by *T. thermohydrosulfuricus, T. brockii* subsp. *finnii, T. pseudethanolicus*, and *T. wiegelii* were tested in this study. None of the tested *Thermoanaerobacter* species could utilize CO (Table 1), but all species could grow and completely convert glucose in the presence of 0, 50, or 100% of CO in the headspace (Table 3, Figures 3, 4). This suggests that CO tolerance is a characteristic present among *Thermoanaerobacter* genus, and not only a property of strain PCO. Carbon recovery at the end of the incubations was nearly 100% for all the growth tests (Table 3). All the tested strains produced the same identified and quantified end products from glucose fermentation, i.e., hydrogen, lactate, acetate, and ethanol, as expected from previous reports (Kim et al., 2001; Lee et al., 2007; Balk et al., 2009; Shaw et al., 2010). Nevertheless, final product concentrations (Figures 3, 4) varied for the different tested strains. Ethanol production was in general less affected by the presence of different CO concentrations. A significant decrease in ethanol production was only observed in the presence of 100% CO and just for two of the five microorganisms tested: strain PCO (*P* = 0.004) and *T. wiegelii* (*P* = 0.002). H_2_ production by all the tested strains decreased significantly, even in the presence of low CO concentrations. In the presence of 100% CO, H_2_ production by the *Thermoanaerobacter* species tested was decreased by 75–95%. Acetate concentration decreased with increasing CO percentage (reduction between 25 and 50%); the exception was strain PCO for which the final acetate concentration was significantly higher in the presence of high CO percentage [acetate final concentration in cultures with 0 and 100% CO were 5.4 ± 0.6 and 8.3 ± 0.5 mM, respectively (*P* = 0.0003)]. These results show the metabolic changes in versatile *Thermoanaerobacter* species upon addition of CO, resulting in a general decrease in H_2_ production. Although none of the tested *Thermoanaerobacter* strains could convert CO, all were able to withstand CO. The lower H_2_ production suggests that CO is inhibiting the hydrogenases of *Thermoanaerobacter* species. Hydrogenases catalyze the oxidation of hydrogen or the reduction of protons. From recent genomic studies, it was confirmed that [Fe-Fe]-hydrogenases, responsible for hydrogen production, are present and well-conserved in all of the *Thermoanaerobacter* species. However, another class of hydrogenases, [Ni-Fe]-hydrogenases, is present only in *T. thermohydrosulfuricus* and *T. wiegelii* (Verbeke et al., 2013; Bhattacharya et al., 2015). [Fe-Fe] or iron-only hydrogenases are known to be more sensitive to CO than [Ni-Fe]-hydrogenases (Diender et al., 2015), which corroborate the results obtained regarding to the effect of CO on hydrogen production from glucose conversion by *Thermoanaerobacter* species. Hydrogenases were shown to be specifically inhibited by carbon monoxide, since CO binds at the active site of the enzyme (Purec et al., 1962; Gutiérrez-Sánchez et al., 2010; Baffert et al., 2011; Matsumoto et al., 2011; Bertsch and Müller, 2015). Genomic analysis of *T. kivui* revealed the presence of genes encoding for carbon monoxide dehydrogenases (CODH) and Ech-hydrogenases complexes (which are responsible for hydrogen production by carboxydotrophic organisms; Hess et al., 2014), although its ability to convert CO was only reported very recently after adaption to increasing concentrations of CO (Weghoff and Müller, 2016). Proper adaption to CO may be the key for achieving CO conversion by *Thermoanaerobacter* species which contain the necessary genomic machinery. Therefore, the high tolerance to CO and the potential of some microorganisms for CO utilization, make the members of *Thermoanaerobacter* genus important for the biotechnological use of syngas/industrial CO-rich gases. Thermophilic microorganisms including members of *Thermoanaerobacter* genus are interesting catalysts for production of biofuels (Carere et al., 2012; Verbeke et al., 2013; Hess et al., 2014; Bhattacharya et al., 2015; Sant'Anna et al., 2015). From this perspective the present study is important as CO is a way to steer the formation of fermentation products. Further research is needed to get a better insight into how at a molecular level carbon monoxide affects product formation in *Thermoanaerobacter* species.

**Table 2:** **Glucose conversion rates by strain PCO under different CO partial pressures**.

**CO (%) (*P*_total_ = 1.7 × 10^5^ Pa)**	**mM glucose consumed · day^−1^**
0	2.91 ± 0.66
25	2.89 ± 0.54
50	2.90 ± 0.46
75	2.78 ± 0.51
100	2.35 ± 0.18

**Table 3:** **Effect of CO partial pressure on glucose conversion and carbon recovery (%) by strain PCO, ***Thermoanaerobacter thermohydrosulfuricus*** (DSM 527^**T**^), ***T. brockii*** subsp. ***finnii*** (DSM 3389^**T**^), ***T. pseudethanolicus*** (DSM 2355^**T**^), and ***T. wiegelii*** (DSM 10319^**T**^)**.

**Strains**	**CO (%)**	**Glucose consumed (mM)**	**Product yield^a^**				**CO_2_ produced^b^(mmol L^−1^_medium_)**	**Carbon recovery^c^(%)**
			**mol H_2_/mol glucose**	**mol acetate/mol glucose**	**mol lactate/mol glucose**	**mol ethanol/mol glucose**		
Strain PCO	0	17.9 ± 1.80	0.22 ± 0.04	0.29 ± 0.04	0.92 ± 0.11	0.39 ± 0.07	12.2 ± 1.2	88.1 ± 9.4
	25	18.0 ± 0.80	0.08 ± 0.01	0.33 ± 0.03	0.93 ± 0.05	0.43 ± 0.02	13.7 ± 0.5	91.2 ± 4.4
	50	18.3 ± 1.24	0.05 ± 0.01	0.35 ± 0.06	0.85 ± 0.14	0.33 ± 0.04	12.3 ± 1.1	82.2 ± 8.8
	75	17.7 ± 1.54	0.06 ± 0.01	0.34 ± 0.06	0.93 ± 0.12	0.36 ± 0.07	12.3 ± 1.4	87.8 ± 9.3
	100	18.1 ± 0.22	<0.02	0.46 ± 0.03	0.78 ± 0.05	0.21 ± 0.02	12.2 ± 0.7	77.1 ± 2.9
*T. thermohydrosulfuricus*	0	17.8 ± 1.00	0.61 ± 0.06	0.38 ± 0.06	0.64 ± 0.09	0.86 ± 0.14	22.2 ± 2.6	102.3 ± 9.0
	50	15.7 ± 0.90	0.40 ± 0.08	0.28 ± 0.04	0.94 ± 0.14	0.58 ± 0.08	13.5 ± 1.3	100.9 ± 9.4
	100	15.2 ± 0.30	0.18 ± 0.01	0.20 ± 0.01	1.21 ± 0.05	0.37 ± 0.06	8.7 ± 1.0	100.0 ± 3.8
*T. brockii* subsp. *finnii*	0	14.2 ± 1.80	0.37 ± 0.05	0.48 ± 0.07	0.85 ± 0.11	0.66 ± 0.19	16.1 ± 2.6	113.7 ± 15.9
	50	14.9 ± 0.90	0.02 ± 0.002	0.32 ± 0.04	0.89 ± 0.06	0.70 ± 0.13	15.3 ± 1.9	112.4 ± 8.2
	100	15.7 ± 0.40	<0.02	0.30 ± 0.05	0.84 ± 0.04	0.66 ± 0.18	15.1 ± 2.9	105.6 ± 7.7
*T. pseudethanolicus*	0	16.6 ± 0.50	0.22 ± 0.02	0.40 ± 0.02	0.72 ± 0.03	0.63 ± 0.22	17.0 ± 3.6	100.7 ± 8.7
	50	17.1 ± 0.60	<0.02	0.26 ± 0.07	0.70 ± 0.06	0.68 ± 0.10	16.1 ± 2.0	91.4 ± 6.1
	100	16.0 ± 1.60	<0.02	0.29 ± 0.04	0.85 ± 009	0.61 ± 0.12	14.6 ± 1.6	97.5 ± 10.7
*T. wiegelii*	0	17.7 ± 1.80	0.90 ± 0.17	0.39 ± 0.108	0.22 ± 0.04	1.45 ± 0.22	32.6 ± 3.2	124.9 ± 14.3
	50	18.5 ± 1.20	0.71 ± 0.12	0.32 ± 0.03	0.26 ± 0.02	1.43 ± 0.13	32.3 ± 1.9	112.8 ± 8.0
	100	17.1 ± 1.70	0.05 ± 0.02	0.25 ± 0.08	0.91 ± 0.21	0.66 ± 0.26	15.5 ± 4.5	99.0 ± 16.7

a *Product yield = (C_P, tf_ − C_P, t0_)/(C_S, t0_ − C_S, tf_); where, C_P_ is product concentration and C_S_ is glucose concentration measure at time zero (t_0_) and at the end of the assay (t_f_). Note: final product concentration was calculated as an average of the plateau of production curves*.

b *Estimated CO_2_ production considering that 1 mol of CO_2_ is produced for each mol of ethanol or acetate formed [a maximum deviation of 20% was obtained when estimating total CO_2_ concentrations from measured CO_2_ concentration in the headspace summed with the correspondent calculated dissolved CO_2_ (using the Henry law)]*.

c *Carbon recovery (CR) = Σ (n_P, tf_ − n_P, t0_)/(n_S, tf_ − n_S, t0_) + n_biomass_; where n is the number of carbon moles in products (P − acetate, lactate, ethanol, and CO_2_) and in glucose (S) and n_biomass_ is the estimated mol of carbon used for biomass growth*.

*For the calculation of n_biomass_, cell density was analyzed photometrically by optical density at 600 nm (OD) and converted to cell dry weight per liter using experimentally determined conversion factors (OD/(g_dry−weight_/L)) for each of the tested strains: 0.78 (strain PCO), 0.89 (T. thermohydrosulfuricus), 1.36 (T. brockii subsp. finnii), 1.04 (T. pseudethanolicus), and 0.86 (T. wiegelii)*.

## Author contributions

JA planned and performed the experiments, data interpretation and wrote the manuscript. MA assisted in the design of the study, participated in data interpretation as well as revisions of the final manuscript. CP assisted in the design of the study, participated in data interpretation as well as revisions of the final manuscript. AS assisted in the design of the study, participated in data interpretation as well as revisions of the final manuscript. DS conceived the study, participated in the planning and coordination of the study, and revised the manuscript. All authors read and gave approval for publication of the manuscript.

### Conflict of interest statement

The authors declare that the research was conducted in the absence of any commercial or financial relationships that could be construed as a potential conflict of interest.
